# Wnt/β-catenin signaling in the development and therapeutic resistance of non-small cell lung cancer

**DOI:** 10.1186/s12967-024-05380-8

**Published:** 2024-06-13

**Authors:** Zixu Zhang, David Westover, Zhantong Tang, Yue Liu, Jinghan Sun, Yunxi Sun, Runqing Zhang, Xingyue Wang, Shihui Zhou, Nigaerayi Hesilaiti, Qi Xia, Zhenfang Du

**Affiliations:** 1 Department of Genetic and Developmental Biology, School of Medicine, Southeast University, Nanjing, 210003 China; 2 High-Throughput Analytics, Analytical Research and Development, Merck & Co. Inc., Rahway, NJ USA; 3 School of Life Science and Technology, Southeast University, Nanjing, 210018 China

**Keywords:** Non-small cell lung cancer, Wnt/β-catenin signaling, Cancer development, Therapeutic response

## Abstract

Wnt/β-catenin signaling is a critical pathway that influences development and therapeutic response of non-small cell lung cancer (NSCLC). In recent years, many Wnt regulators, including proteins, miRNAs, lncRNAs, and circRNAs, have been found to promote or inhibit signaling by acting on Wnt proteins, receptors, signal transducers and transcriptional effectors. The identification of these regulators and their underlying molecular mechanisms provides important implications for how to target this pathway therapeutically. In this review, we summarize recent studies of Wnt regulators in the development and therapeutic response of NSCLC.

**Supplementary Information:**

The online version contains supplementary material available at 10.1186/s12967-024-05380-8.

## Introduction

Lung cancer is one of the leading causes of cancer death worldwide, of which 80% to 85% is non–small cell lung cancer (NSCLC). Lung adenocarcinoma (LUAD) accounts for approximately 85% of NSCLC diagnoses, with lung squamous cell cancer (LUSC) accounting for approximately 15%, based on histological classification [1]. The 5-year survival rate for NSCLC is only 26.5% because the disease is usually metastatic at diagnosis. Metastatic NSCLC is generally incurable, as it almost always develops therapeutic resistance after an initial response [2].

The Wingless/integrase-1 (Wnt) family is a type of secreted glycoproteins which interacts with transmembrane receptors and contributes to the development and differentiation of multiple organs, including lung [3]. Wnt family proteins, of which there are 19 in humans, function as ligands to conduct a signal from the cell surface through the cytoplasm to the nucleus, thereby regulating expression of a coordinated sets of genes involved in multiple biological processes. Based on whether it relies on β-catenin for transcription activation, Wnt signaling pathways can be divided into the canonical pathway, namely Wnt/β-catenin signaling pathway, and non-canonical pathways, including Wnt/PCP pathway and Wnt/Ca^2+^ pathway [4]. Abnormal alterations of the Wnt/β-catenin pathway by its regulators contribute to the development and therapeutic responses of NSCLC [5].

**Fig. 1:**
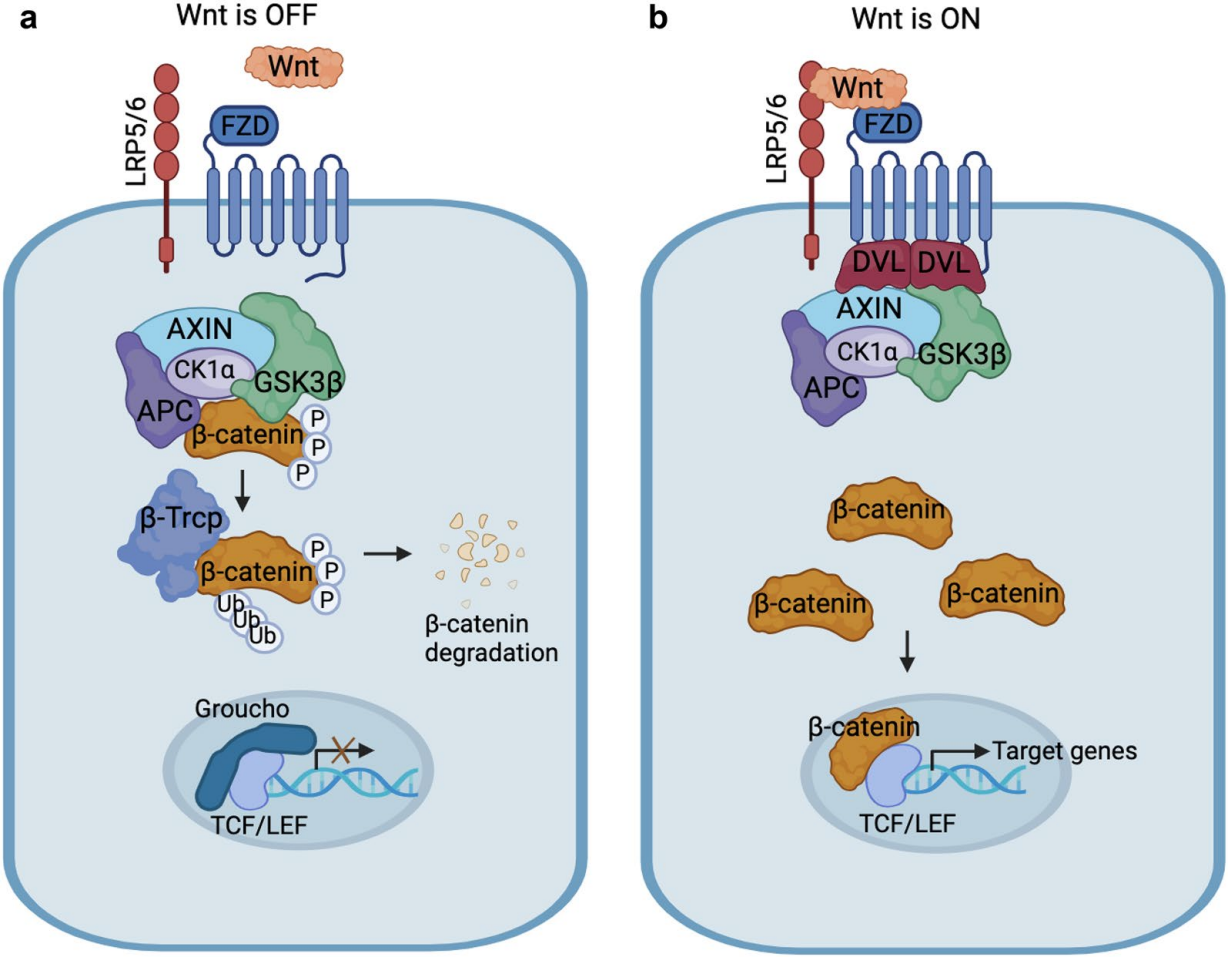
An overview of Wnt/β-catenin signaling pathway. **a** In the absence of the Wnt signal, cytosolic β-catenin is phosphorylated by kinases CK1α and GSK3β with the help of scaffolding proteins AXIN and APC. Phosphorylation of β-catenin leads to its ubiquitylation and subsequent proteasomal degradation. **b** Wnt ligands bind FZD and LRP5/6 receptors on the cell surface. Subsequent phosphorylation of LRP5/6 and recruitment of signal transducers DVL and AXIN to the Wnt-bound receptors facilitate inhibition of GSK3β activity. This inhibition blocks phosphorylation and degradation of β-catenin, leading to β-catenin accumulation in the cytoplasm and translocation into the nucleus. In the nucleus, β-catenin interacts with TCF/LEF transcription factors to activate Wnt target genes

β-catenin functions in a dual role, either as the most important nuclear effector of Wnt/β-catenin signaling, or as a cytoskeletal junction protein that maintains cell adhesion, which is critical for cadherin-based adherens junctions (AJs). These dual functions are carried out based on the transcriptional pool and the adhesive pool of β-catenin [6]. In the transcriptional pool, Wnt ligands initiate a Wnt/β-catenin signaling cascade, which involves the translocation of β-catenin from cytoplasm to nucleus and activation of target genes via T cell factor (TCF)/lymphoid enhancer-binding factor (LEF) family of transcription factors (Fig. 1). In the absence of Wnt ligands, pathway signaling is inactivated by a “destruction complex” comprised of the tumor suppressor Adenomatous Polyposis Coli (APC), the scaffolding protein AXIN, casein kinase 1α (CK1α) and glycogen synthase kinase 3 β (GSK-3β) [7]. Cytoplasmic β-catenin is sequestered in this destruction complex and sequentially phosphorylated by CK1α at Ser45 and GSK3β at Ser33/Ser37/Thr41, respectively [8]. Phosphorylated β-catenin is then recognized by E3 ubiquitin ligase β-Trcp and ubiquitinated for proteasomal degradation [7]. Without β-catenin in the nucleus, Groucho family transcription repressors bind to TCF/LEF transcription factors and inhibit the transcription of Wnt target genes. When present, Wnt ligands bind to the Frizzled (FZD) receptor family and a member of the low-density lipoprotein receptor–related protein (LRP) family, LRP5 or LRP6, to form FZD-LRP5/6 complexes. These complexes recruit the signal transducer Dishevelled (DVL) to the membrane for phosphorylation and oligomerization [9]. Phosphorylated DVL recruits AXIN and inhibits its interaction with other components of the destruction complex, thereby preventing proteasomal degradation of β-catenin [10]. Thus, the concentration of β-catenin will increase in the cytoplasm, translocating to the nucleus and forming a co-transcriptional complex with TCF/LEF, which activates the transcription of the downstream target genes which will regulate cell fate, migration, and tissue configuration [4].

In the adhesive pool, β-catenin acts as the core component of the AJs and regulates the aggregation of cadherin by directly binding to the cytoplasmic domain of E-cadherin and the actin-binding protein α-catenin, maintaining cell–cell junctions, tissue structural integrity, and homeostasis [11]. The canonical function of the AJs is to initiate and stabilize cell–cell adhesion between neighboring cells and to modulate actin dynamics at the cortical level, and dysfunctions of AJs contribute to cancer progression [12].

Epithelial‑mesenchymal transition (EMT) comprises an essential biological process during which cells fail to maintain epithelial cell polarity and acquire a mesenchymal phenotype, thus facilitating invasion and metastasis. During the early phase of EMT, loss of apical–basal polarity is often the first event to be observed and can lead to the destabilization of adhesion complexes, including AJs at the lateral membrane [13]. Wnt/β-catenin signaling is one of the most important pathways involved in the regulation of EMT. Wnt/β-catenin signaling exerts its effect on EMT through targeting and activating EMT-transcription factors SNAIL, SLUG, and TWIST which will regulate the expression of E-cadherin and N-cadherin. Wnt/β-catenin signaling can also impact EMT through AJs by other Wnt/β-catenin-targeted genes such as *MMP7* and *TIAM1* [14].

Wnt regulators influence Wnt/β-catenin signaling at both the transcriptional and translational level, with regulators identified that act on ligands, receptors, signal transducers and transcriptional effectors. These regulators might be proteins, microRNAs (miRNAs), long noncoding RNAs (lncRNAs), or circular RNAs (circRNAs) [5]. miRNAs contain 20–25 nucleotides which repress translation of targeted mRNAs or target mRNA degradation [15]. LncRNAs are RNA transcripts longer than 200 nucleotides, and most of them do not encode peptides. LncRNAs encompass natural antisense transcripts, overlapping transcripts and intronic transcripts, which regulate gene expression through a variety of different mechanisms, including acting as molecular scaffolds that ‘guide’ chromatin-modifying enzymes, competing endogenous RNAs (ceRNAs) that ‘sponge’ miRNAs or proteins, facilitating or inhibiting long-range chromatin interactions, or functioning through the act of transcription itself [16]. CircRNAs are a class of single-stranded noncoding RNAs in circular form through non-canonical splicing or back-splicing manner. CircRNAs can serve as miRNA sponge in which circRNAs bind directly to the targeted miRNAs to inhibit miRNA activity, or affect alternative splicing through RNA-mediated interaction, or interact with RNA-binding proteins as protein scaffolds or antagonists [17].

Based on functional effect, Wnt regulators can be classified as positive and negative regulators. The upregulation of positive regulators and downregulation of negative regulators will promote the activation of Wnt/β-catenin signaling pathway. Aberrant Wnt regulator expression and signaling have been identified in lung cancer cell lines, animal models, and human NSCLC tissues [18–23]. Modulation of these regulators provide potential treatment strategies for patients with NSCLC, and many agents that suppress Wnt/β-catenin signaling also inhibit NSCLC cell lines [24, 25]. In this review, we mainly focus on the recent studies of regulators identified in Wnt/β-catenin signaling implicated in development and therapeutic responses of NSCLC.

## Aberrant alterations of Wnt components in NSCLC

In humans, the complexity and specificity of Wnt signaling is achieved partially through 19 Wnt ligands [4]. The aberrant expression of most Wnt ligands have been found to closely correlate to the occurrence and progression of NSCLC, and are the thus potential biomarkers and drug targets for the diagnosis, prognosis, and treatment of NSCLC [26]. Overexpression of *WNT2B*, *WNT3A* and *WNT5A* has been found to associate with NSCLC [27, 28] (Table 1). FZD family members are a type of seven-pass transmembrane receptor (FZD1-FZD10) that belong to atypical G protein-coupled receptors (GPCRs). Specifically, FZD2 expression was found to associate with the prognosis of LUAD [29], and promoter CpG methylation of *FZD2* might be related to the prognosis of LUSC [30]. Abnormal expression of many FZDs (FZD3, FZD8 and FZD9) is associated with the development of NSCLC [3]. It has been observed that patients with early-stage NSCLC carrying the SNP rs10898563 in *FZD4* showed a significant increase in recurrence and mortality risk [31], and FZD4 expression might be associated with the prognosis of LUAD [29]. Knockdown of *FZD8* by shRNA sensitized the lung cancer cells to chemotherapy [32]. *FZD10* methylation was found to possibly relate to the prognosis of patients with LUSC [30] (Table 1).

**Table 1:** Wnt components which have been reported to associate with NSCLC

Components	Alterations	Specimen	Clinical relevance	References
*WNT2B*,* WNT5A*	Overexpression	NSCLC tissues	M2 and M1 tumor‑associated macrophages	[27]
*WNT3A*	Overexpression	LUAD tissues	Poorer survival	[28]
*FZD2*	Underexpression	NSCLC	Prognosis	[29]
*FZD2*	Methylation	LUSC	Prognosis	[30]
*FZD3*	Overexpression	NSCLC	Not determined	[33]
*FZD4*	SNP rs10898563	Early-stage NSCLC	Recurrence and death risk	[31]
	Underexpression	NSCLC tissue	Prognosis	[29]
*FZD8*	Overexpression	NSCLC	Not determined	[32, 255]
	Overexpression	A549 and A427 cell line	Cell proliferation	[32, 255]
*FZD9*	Underexpression	NSCLC tissue	Not determined	[256]
*FZD10*	Methylation	LUSC patients	Prognosis	[30]
*LRP5*	Underexpression	LUSC	Occurrence risk	[33]
	SNP (rs3736228 and rs64843)	LUSC/NSCLC	Occurrence risk	[34]
*LRP6*	SNP	LUSC/NSCLC	Occurrence risk	[35]
*DVL1*	Overexpression	Brain metastases from LUAD	Poor prognosis	[36]
	Overexpression	NSCLC	Clinicopathologic characteristics and poor prognosis	[37]
*DVL3*	Overexpression	Brain metastases from LUAD	Poor prognosis	[36]
*AXIN1*	DNA methylation	H446 and H157 cell line	Radiosensitivity	[38]
	DNA methylation	Lung cancer tissue	Clinical characteristics	[39]
	Underexpression	Micropapillary-predominant LUAD	Invasion	[40]
*AXIN2*	SNP (intronic 1712 + 19 variant, GG)	Indian patients with LUAD	Increased death risk	[41]
	SNP (intronic 1712 + 19 variant, GT)	Indian patients with LUAD	Decreased death risk	[42]
	rs2240308 (148 C/T) and 1365 C/T	Chinese patients with LUAD	Decreased cancer susceptibility	[43, 44]
	DNA methylation	NSCLC	Prognosis and histological subtyping	[45]
*CK1α (CSNK1A1)*	DNA methylation	NSCLC	Prognosis and histological subtyping	[45]
	Overexpression	NSCLC	Poor prognosis	[46]
*CTNNB1*	rs1880481 (AC/AA)	NSCLC	Decreased risk of bone metastasis, longer median progression free survival time	[257]
TCF-4	The 10th exon partial deletion	TCGA LUAD	Better overall survival	[258]

For LRP5/6 receptor, *LRP5* expression has been shown to be decreased in LUSC [33]. SNPs in *LRP5* were found to associate with an higher risk of NSCLC (SNP rs3736228) and LUSC (SNP rs64843) [34]. SNP rs10845498 on *LRP6* is associated with a lower risk of LUSC, whereas *LRP6* rs6488507 is associated with higher risk of NSCLC in tobacco smokers [35]. For Dishevelled (DVL), upregulated expression of *DVL1* and *DVL3* was found in brain metastases from LUAD [36]. Overexpression of *DVL1* is associated with unfavorable prognosis of patients with NSCLC [37] (Table 1).

**Fig. 2:**
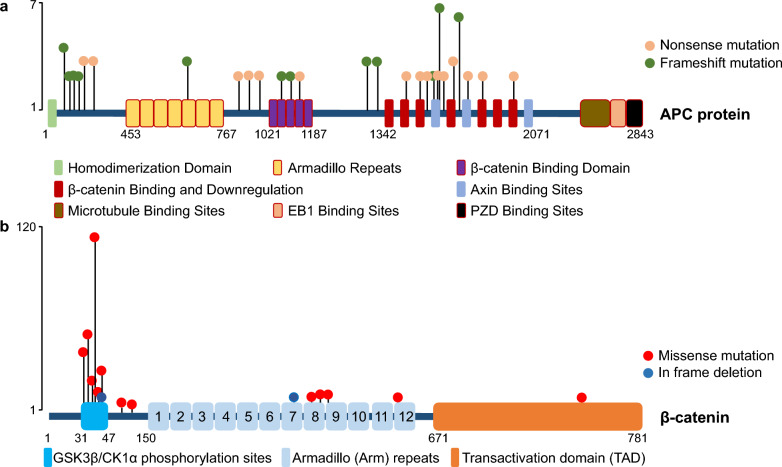
*APC* and *CTNNB1* mutations identified in NSCLC from GENIE datasets (GENIE 14.0-public, n = 26,473). **a** The recurrent truncating *APC* mutations (n ≥ 2) in NSCLC were shown on the schematic structure. The truncating mutations include nonsense mutations and frameshift mutations. **b** The recurrent *CTNNB1* mutations (n ≥ 2) were shown on the schematic structure. β-catenin is sequestered in the destruction complex and sequentially phosphorylated by CK1α at Ser45 and GSK3β at Ser33/Ser37/Thr41, respectively. The truncating mutations were not included in this study

For components of the destruction complex, *AXIN1* methylation was found to correlate with radiosensitivity of lung cancer cells and clinical features of NSCLC [38, 39] (Table 1). Downregulation of *AXIN1* expression was found in micropapillary-predominant LUAD, especially in cases with lymph node invasion, indicating diminished *AXIN1* expression may affect the invasiveness of LUAD [40]. The intronic *AXIN2* 1712 + 19 variant exhibited increased mortality in Indian LUAD patients with GG genotype [41], while the heterozygous (GT) genotype showed a decreased risk of mortality [42]. *AXIN2* 148 C/T and 1365 C/T variants might be associated with reduced cancer susceptibility in Chinese NSCLC patients [43, 44]. Aberrant promoter methylation of *AXIN2* was observed in NSCLC, and might be related to prognosis and histological subtyping of NSCLC [45]. High expression of *CSNK2A1*, which encodes CK1α, is an independent prognostic factor of poor survival for NSCLC patients [46] (Table 1). *APC* and *CTNNB1* mutations were also found in NSCLC (Fig. 2). In NSCLC, *APC* mutations are mostly loss-of-function truncating mutations which are evenly distributed across *APC* gene (Fig. 2a; Supplementary Table 1); *CTNNB1* mutations are mostly gain-of-function point mutations that mainly concentrate on the GSK3β/CK1α phosphorylation sites (Fig. 2b; Supplementary Table 2). The mutations on phosphorylation sites prevent the phosphorylation of β-catenin and so escape from E3 ubiquitin ligase β-Trcp and subsequent proteasomal degradation, thus leading to the accumulation of β-catenin and elevated Wnt/β-catenin signaling [47].

## The positive regulators of Wnt/β-catenin signaling

Many positive regulators have been identified which act on Wnt ligands, receptors, transducers, components of β-catenin destruction complex, and β-catenin. These regulators might be overexpressed, amplified, or mutated in NSCLC cells.

### Wnt ligands

The expression of multiple Wnt ligands have been found to be upregulated in NSCLC, including *WNT1*, *WNT2B*, *WNT3A* and *WNT5A* (Table 2). *WNT1* transcriptional expression was upregulated by PHF8, a histone demethylase. Higher *PHF8* expression was found in NSCLC and correlated with poorer overall survival in NSCLC patients. Mechanistically, PHF8 increases *WNT1* transcription by targeting the promoter region of *WNT1* and so removing the histone markers there [48]. *WNT2B* expression was upregulated by the RNA helicase DDX56 [49] and lncRNA *RPPH1* [50]. *DDX56* overexpression was found in LUSC and negatively associated with recurrence-free survival in LUSC patients. DDX56 increased the transcription of the target gene *WNT2B* through the degradation of primary miR-378a [49]. *RPPH1* overexpression was negatively associated with disease progression and overall survival. Mechanistically, lncRNA *RPPH1* promoted NSCLC progression through miR-326/*WNT2B* axis as *WNT2B* is a target gene of miR-326 [50]. Another lncRNA, AL139294.1, promotes *WNT5A* expression and oncogenic activity through suppression of miR-204-5p [51]. *WNT3A* expression was upregulated by PITX2 [52], ASPM [53], GOLPH3 [54], ALDOC [55] and FAIM2 [56] (Table 2). PITX2 binds directly to the promoter of *WNT3A* and upregulated its transcriptional expression. High *PITX2* expression was found in LUAD and correlated with worse prognosis [52]. GOLPH3 is a peripheral membrane protein localized to the trans-Golgi. High expression of *GOLPH3* was found in NSCLC tissues and was associated with clinicopathologic characteristics. GOLPH3 interacts with CKAP4 and increases the secretion of exosomal WNT3A, leading to a cancer stem cell (CSC)-like phenotype and metastasis in NSCLC [54]. *WNT5A* expression was found to be upregulated by PTS [57], circVAPA [58], E2F1 [59] and ATF4 [60] (Table 2). Higher PTS level was found in LUAD and correlated with late clinical stages and poor survival [57]. circVAPA acted as a ceRNA to up-regulate WNT5A by sponging miR-876-5p and thus activating Wnt/β-catenin signaling [58]. Intriguingly and perhaps paradoxically, WNT5A has also been reported to inhibit Wnt/β-catenin signaling in EGFR-mutant cells. In this scenario, E2F1-mediated repression of *WNT5A* expression promotes brain metastasis EGFR-mutant NSCLC, and high expression of *E2F1* was negatively correlated with the expression of *WNT5A* and associated with poor outcomes in NSCLC [59].

**Table 2:** Positive Wnt regulators recently reported to involve in NSCLC

Name	Alterations	Target	Specimen	Clinical relevance	References
PHF8	Overexpression	*WNT1* promoter	NSCLC tissues and cell lines	Poor OS	[48]
DDX56	Overexpression	miR-378a/miR-378a-3p/*WNT2B*	LUSC tissues	Poor prognosis	[49]
lncRNA RPPH1	Overexpression	miR-326/*WNT2B* axis	NSCLC tissues and cell lines	Poor prognosis	[50]
PITX2	Overexpression	Promoter of *WNT3A*	LUAD	Clinicopathologic characteristics and poor prognosis	[52]
ASPM	Overexpression	*WNT3A*/GSK3β/β-catenin	NSCLC tissues	Poor overall survival	[53]
FAIM2	Overexpression	WNT3A/β-catenin	NSCLC tissues and bone metastasis	Clinicopathologic characteristics and poor prognosis	[56]
ALDOC	Overexpression	UBE2N/WNT3A/β-catenin	NSCLC tissues	Clinicopathologic characteristics and poor prognosis	[55]
GOLPH3/CKAP4	Overexpression	Exosomal WNT3A	NSCLC tissues and cell lines	Clinicopathologic characteristics and poor prognosis	[54]
PTS	Overexpression	WNT5A/GSK-3β/β-catenin	LUAD tissues	Poor OS	[57]
circVAPA	Overexpression	miR-876-5p/WNT5A axis	NSCLC tissues and cell lines	Unknown	[58]
E2F1	Overexpression	WNT5A (which inhibits β-catenin activity)	NSCLC tissues	Poor OS	[59]
ATF4	Overexpression	WNT5A/GSK3β/β-catenin	A549, H1299, and LK2 cells	Unknown	[60]
LINC00942	Overexpression	miR-5006-5p/FZD1	LUAD from TCGA	Poor prognosis	[61]
Hsa_circ_0017109	Overexpression	miR-671-5p/FZD4/β-catenin axis	NSCLC tissues and cell lines	Poor prognosis	[62]
Ninj1	Overexpression	FZD2-LRP6 assembly	NSCLC tissues and cell lines	Poor prognosis	[65]
ENO1	Overexpression	p-LRP5/6	NSCLC tissues and metastatic NSCLC cell lines	Poor OS, and advanced TNM and metastatic stage	[19]
TRIP13	Overexpression	LRP6	NSCLC tissues	Advanced tumor stage and poor OS	[64]
CD248	Overexpression	Interaction of IGFBP4 and LGALS3BP with LRP6	Fibroblasts and pericytes	Poor survival	[66]
USP46	Amplification	Deubiquitylation of LRP6	LUSC	N.D	[67]
miR-1254	Overexpression	3′UTR of *SFRP1*	NSCLC tissues and cell lines	Unknown	[69]
Exosomal-miR-1260b	Overexpression	*SFRP1*	LUAD tissues and plasma	Unknown	[71]
HNRNPA2B1	Overexpression	m6A mediate pri-miR-106b/miR-106b-5p/SFRP2	LUAD CSCs (A549 and H1975)	Poor prognosis	[73]
METTL3	Overexpression	N6-methyladenosine modifying *SFRP2*	NSCLC tissues	Unknown	[74]
RYR2	Mutation	DKK1	NSCLC samples from UCSC Xena database	Clinical characteristics and better prognosis	[75]
LINC00467	Overexpression	*DKK1* promoter	TCGA LUAD samples	Poor OS	[76]
Cytosol-localized TMEM88	Overexpression	DVLs	NSCLC tissues	Clinicopathologic characteristics and poor survival	[78]
CtBP2	Overexpression	DVL1	NSCLC tissues and cell lines	Clinicopathologic characteristics and poor prognosis	[77]
PWP1	Overexpression	DVL2	NSCLC tissues	Clinicopathologic characteristics and poor prognosis	[79]
LINC00673-v4	Overexpression	DVL phosphorylation	TCGA LUAD	Lymph node metastasis and poor prognosis	[80]
AQP3	Overexpression	Transcription of *GSK3B* and *CTNNB1*	NSCLC A549 cell line	Unknown	[81]
miR-19a/19b	Overexpression	3′UTR of *GSK3B*	Sulforaphane treated lung CSCs (A549 and H1299)	Unknown	[82]
miR-1246	Overexpression	3′-UTR of *GSK3B*	Serum of NSCLC patients	Unknown	[83]
miR-1275	Overexpression	3′-UTR of *DKK3*, *SFRP1* and *GSK3B*	LUAD tissues and cell lines	Tumor progression and poor prognosis	[84]
lncRNA JPX/miR-33a-5p/Twist1 axis	Overexpression	GSK3β/nuclear β-catenin	NSCLC tissues and cells	Clinicopathologic characteristics	[86]
PHLDA3	Overexpression	GSK3β	LUAD tissues	Clinicopathologic characteristics and poor prognosis	[87]
miR-582-3p	Overexpression	3′-UTRs of *AXIN2*, *DKK3* and *SFRP1*	NSCLC cell lines and tissues	Recurrence and poor prognosis	[85]
LL-37	Overexpression	Axin2	NSCLC tissues and sera	Poor prognosis	[259]
Zbed3	Overexpression	AXIN-GSK3β complex	NSCLC	Unknown	[90]
YTHDF2	Overexpression	*AXIN1*	LUAD from TCGA	Poor OS	[23]
APEX1	Overexpression	Aberrant alternative splicing of *AXIN1*	NSCLC from TCGA	Unknown	[92]
GTPBP2	Overexpression	AXIN	NSCLC tissues	Clinicopathologic characteristics and poor prognosis	[91]
RIF1	Overexpression	PP1-AXIN interaction	NCSLC tissues	Poor prognosis	[93]
DLX6-AS1-encoded peptide	Overexpression	AXIN2/β-catenin	NSCLC	Unknown	[94]
miR-4326	Overexpression	3′UTR of *APC2*	Lung cancer from TCGA	Unknown	[95]
miR-3607	Overexpression	3′UTR of *APC*	Lung cancer from TCGA	Unknown	[96]
miR-4739	Overexpression	APC and DKK3	"Driver gene-negative" NSCLC tissues and cell lines	Clinicopathologic characteristics and poor prognosis	[97]
RNF115	Overexpression	APC ubiquitination	LUAD from TCGA	Poor OS	[98]
FLVCR1-AS1	Overexpression	Transcription of *CTNNB1*	NSCLC from TCGA	Unknown	[100]
LINC01006	Overexpression	MicroRNA 129-2-3p/*CTNNB1* ceRNA Axis	LUAD tissues and cell lines	Unknown	[101]
lncRNA SNHG11	Overexpression	miR-4436a/*CTNNB1* ceRNA axis	NSCLC tissues	Clinicopathologic characteristics and poor prognosis	[102]
TET	Loss of function mutations	DNA methylation of Wnt-related antagonists (*LRP4* and *CTNNB1*)	LUAD from TCGA	Poor OS in GEMMs	[103]
eIF3a	Overexpression	YY1 mediated transcriptional activation of *CTNNB1*	NSCLC tissues	Poor prognosis	[104]
WSB2	Overexpression	Transcription of *CTNNB1*	Xuanwei lung cancer (mostly LUAD) tissues and cell lines	Advanced tumor stages	[105]
CIRP	Overexpression	3′- and 5′-UTRs of *CTNNB1* mRNA	NSCLC tissues	Poor prognosis	[106]
miR-214	Overexpression	3′ UTR of *CTNNB1* mRNA	NSCLC tissues	Clinicopathologic characteristics and poor OS	[107]
LINC00514	Overexpression	β-catenin	NSCLC tissues and cell lines	Poor prognosis	[109]
lncRNA ITGB1-DT	Overexpression	ITGB1-DT/ITGB1/β-catenin/MYC positive feedback loop	LUAD tissues	Advanced tumor stages and poor prognosis	[110]
lncRNA UPLA1	Overexpression	Desmoplakin/β-catenin	LUAD tissues	Clinicopathologic characteristics and poor prognosis	[21]
RNASEH1-AS1	Overexpression	miR-516a-5p/FOXK1/β-catenin axis	LUAD and LUSC tissues	Poor prognosis	[111]
Circ-EIF3I	Overexpression	miR-1253/NOVA2/β-catenin	Lung cancer tumor tissues and cell lines	Poor prognosis	[112]
circZSWIM4	Overexpression	miR-370-3p/FOXM1/β-catenin	LUAD cell lines	Unknown	[113]
KDM2B	Overexpression	EZH2/PKMYT1/β-catenin axis	NSCLC patients	Poor prognosis	[114]
SETD1A	Overexpression	NETA1/EZH2/β-catenin axis	NSCLC tissues	Poor prognosis	[115]
EHD1	Overexpression	EHD1/14-3-3ζ/β-catenin/c-Myc positive feedback circuit	NSCLC tissue	Maximum standard uptake value (SUVmax) in ^18^F-FDG PET/CT scans	[116]
TRIM27	Overexpression	SIX3/β-catenin	NSCLC tissues	Clinicopathologic characteristics and poor OS	[117]
HORMAD1	Overexpression	AKT/β-catenin	LUAD tissues	Poor prognosis	[118]
lncRNA PKMYT1AR/ miR-485-5p /PKMYT1 axis	Overexpression	β-TrCP1 mediated ubiquitin degradation of β-catenin	NSCLC tissues and cell lines	Poor prognosis	[99]
FABP7	Overexpression	Ubiquitin-proteasomal degradation of β-catenin	NSCLC metastatic tissues	Poor prognosis	[121]
USP5	Overexpression	β-catenin ubiquitination	TCGA-LUAD	Poor prognosis	[122]
FOXH1	Overexpression	β-catenin	NSCLC from TCGA	Poor prognosis	[123]
LRP8	Overexpression	β-catenin	NSCLC tissues and cell lines	Clinicopathological characteristics and poor prognosis	[124]
CBX4	Overexpression	β-catenin	LUAD tissues	Poor prognosis	[125]
SMEK1	Overexpression	β-catenin	LUAD tissues	No significance	[126]
JAML	Overexpression	β-catenin	LUAD tissues	TNM stages	[127]
ERCC6L	DNA amplification and low methylation	β-catenin	LUAD tissues	Clinicopathological characteristics and poor prognosis	[128]
NOVA1	Overexpression	β-catenin	NSCLC tissues	Clinicopathologic characteristics and poor prognosis	[129]
SETDB1	Overexpression	β-catenin	NSCLC samples	Tumor grade	[130]
HMGB1	Overexpression	β-catenin	Lung cancer tissues	Tumor size and TNM stage	[131]
DEPDC1B	Overexpression	β-catenin	NSCLC tissues and cell lines	Poor OS	[132]
MORC2	Overexpression	Active β-catenin	NSCLC tissues	Clinicopathologic characteristics and poor prognosis	[133]
PLAC8	Overexpression	AKT/active β-catenin	Lung cancer tissues and plasma	Unknown	[134]
CCDC85B	Overexpression	AKT/active β-catenin	Cytoplasm of NSCLC tumor cells	Clinicopathologic characteristics	[135]
KIF26B	Overexpression	AKT/active β-catenin	LUAD and LUSC tissues	Poor OS	[136]
TMED3	Overexpression	AKT/active β-catenin	LUAD and LUSC from TCGA	Poor prognosis	[137]
ARHGEF40	Overexpression	AKT/active β-catenin	NSCLC tissues	Clinicopathologic characteristics and poor prognosis	[88]
Tumor-Intrinsic PD-L1	Overexpression	Active β-catenin	NSCLC tissues	Clinicopathologic characteristics	[138]
S100A4	Overexpression	Phosphorylated β-catenin (Ser552)	LUAD tissues	Tumor size and advanced tumor grades	[139]
lncRNA CBR3-AS1	Overexpression	Nuclear β-catenin	LUAD tissues	Poor OS	[140]
LINC00669	Overexpression	Nuclear β-catenin and protein level of TCF-1	NSCLC tissues	Poor OS and progress free survival	[141]
MEF2D	Overexpression	NUSAP1/accumulation of nuclear β‑catenin	NSCLC tissues and cell lines	Clinicopathologic characteristics and poor OS	[142]
ASNS	Mutation	Accumulation of nuclear β-catenin	NSCLC tissues	Metastasis and poor prognosis	[143]
SRPK1	Overexpression	Accumulation of nuclear β-catenin	NCSLC tissues	Clinicopathologic characteristics and poor prognosis	[144]
Pygo2	Overexpression	Nuclear β-catenin	Lung cancer tissues and cell lines	Unknown	[145]
WDR74	Overexpression	Accumulation of nuclear β-catenin	LUAD and LUSC tissues	NSCLC progression and poor prognosis	[146]
DSTN	Overexpression	Accumulation of nuclear β-catenin	LUAD tissues	Clinicopathologic characteristics and poor prognosis	[147]
SOX9	Overexpression	Accumulation of nuclear β-catenin	NSCLC tissues	TNM stage	[148]
Nuclear E-cadherin	Overexpression	β-catenin/TCF4 complex	Lung cancer tissues	Prognostic value	[149]
Pygo1	Overexpression	β-catenin/TCF4 complex	Early-stage NSCLC tissues	Poor OS	[150]
FOXP3	Overexpression	β-catenin/TCF4 complex	NSCLC tissues	Poor OS and recurrence-free survival	[151]
TRIB3	Overexpression	β-catenin/TCF4 complex	LUAD tissues	TNM stages, lymph node metastasis and poor prognosis	[152]

*N.D.* not determined, *CDX* cell line derived xenograft, *PDX* patient derived xenograft, *KP* *LSL-Kras*^*G12D*^, *P53*^*loxp/loxp*^ mouse model, *OS* overall survival

### Wnt receptors

Many positive regulators act on Wnt receptors by multiple mechanisms in NSCLC. *FZD1* expression is upregulated by *LINC00942* in LUAD (Table 2). Higher expression of *LINC00942* was found in LUAD tissues and associated with poorer survival. Mechanically, *LINC00942* functioned as a ceRNA which targets miR-5006-5p and increases the expression of its direct target *FZD1* [61]. *FZD4* expression was found upregulated by circRNA *hsa_circ_0017109*. Upregulation of this circRNA was found in NSCLC tumor and cell lines. *Circ_0017109* regulated *FZD4* expression by targeting miR-671-5p and finally activated Wnt/β-catenin signaling [62].

The phosphorylation of LRP5/6 recruits AXIN and GSK3β to its phosphorylated sites, leading to the disassembly of β-catenin destruction complex. As a result, β-catenin accumulate in cytoplasm which finally translocate to the nucleus and enhance the transcription of targeted genes [63]. LRP5/6 phosphorylation is upregulated by ENO1 [19] (Table 2), which is a metabolic enzyme involved in the synthesis of pyruvate. ENO1 also decreased GSK3β activity, inactivated the β-catenin destruction complex and ultimately upregulated β-catenin. Higher expression of *ENO1* was found in metastatic lung cancer cell lines and patients, and associated with worse overall survival of patients with NSCLC [19]. LRP6 can directly interact with TRIP13 [64] and NINJ1 [65] (Table 2). TRIP13 is an ATPase which is highly expressed in NSCLC, correlating with advanced tumor stage and poor patient survival. TRIP13 promotes NSCLC cell proliferation and invasion through activating Wnt/β-catenin signaling [64]. NINJ1 is a 17-kDa homophilic cell adhesion molecule located in the cell membrane. *NINJ1* overexpression was found to associate with poor prognosis in patients with NSCLC. Mechanistically, NINJ1 forms an assembly with LRP6 and FZD2, resulting in transcriptional upregulation of Wnt downstream target genes [65]. CD248 inhibits the interaction between LRP6 and Wnt repressors IGFBP4 and LGALS3BP, increasing Wnt/β-catenin signaling in pericytes to promote angiogenesis and tumor growth in lung cancer [66]. Ubiquitylation also participate into the regulation of LRP6. USP46 is a deubiquitylase which form complex with the catalytic USP46 and the WDR40-repeat proteins, WDR20 and UAF1. This complex increases the steady-state level of cell surface LRP6 and facilitates the assembly of LRP6 into signalosomes through the removal of sterically hindering ubiquitin chains. Alterations in USP46 mostly consisted of amplification and were commonly observed in LUSC [67].

SFRP family contains 5 members (SFRP1-5) and negatively regulate Wnt/β-catenin signaling by competing with FZD receptors to bind Wnt ligands extracellularly [68]. Dickkopf (DKK) family contains 3 members (DKK1-3) which negatively regulate Wnt/β-catenin signaling by preventing the interaction of Wnt ligands with LRP5/6 [68]. Inhibition of these negative regulators can promote the activation of Wnt/β-catenin signaling. Expression was found to be downregulated by miR-1254 [69], Rab37 [70] and exosomal-miR-1260b [71] (Table 2). miR-1254 suppresses *SFRP1* expression through binding to its 3′ UTR. miR-1254 was upregulated in lung cancer tissues and promoted lung cancer cell proliferation [69]. Exosomal-miR-1260b was highly expressed in plasma of patients with LUAD and potentiated Wnt/β-catenin signaling by suppressing *SFRP1* expression [71]. N6-methyladenosine (m6A) methylation is a key regulatory mechanism for gene expression and involved in multiple biological processes including cancer development [72]. *SFRP2* expression was found to be regulated by m6A methylation through m6A reader HNRNPA2B1 [73] and writer METTL3 [74] (Table 2). HNRNPA2B1 regulates the maturing of miR-106b-5p through m6A methylation, so that miR-106b-5p targeted and suppressed *SFRP2*, activating Wnt/β-catenin signaling, and thus to aggravate the stemness and progression of LUAD [73]. *SFRP2* expression was found to be negatively regulated by METTL3, which subsequently activated the Wnt/β-catenin signaling pathway in NSCLC [74]. *DKK1* expression was found to be downregulated by RYR2 [75] and LINC00467 [76] (Table 2). *RYR2* mutation prolongs survival of NSCLC patients via down-regulation of *DKK1* expression [75]. LINC00467 promotes the development of LUAD by epigenetically silence of *DKK1* [76].

### Wnt transducers

The expression of signal transducer DVLs have been found to be upregulated by CtBP2 [77], TMEM88 [78], PWP1 [79] and lncRNA *LINC00673-v4* [80] (Table 2). CtBP2 directly interacts with DVL1 and activates Wnt/β-catenin signaling in NSCLC cells [77]. Cytoplasmic TMEM88, rather than the membrane-localized TMEM88, promotes invasion and metastasis in NSCLC cells by binding DVLs. Higher expression of cytoplasmic TMEM88 was found to significantly associate with poorer clinical characteristics and inferior survival in patients with NSCLC [78]. PWP1 interacts with DVL2 and activates Wnt/β-catenin signaling pathway. PWP1 overexpression was found in NSCLC and correlates with poor clinical features [79]. LINC00673-v4 overexpression was associated with adverse clinical outcome. Mechanically, LINC00673-v4 enhanced the interaction between DDX3 and CK1ε and thus upregulated the phosphorylation of DVLs [80].

### β-Catenin destruction complex

The β-catenin destruction complex consists of GSK-3β, APC, AXIN and two CK1α. *GSK3B* transcription was downregulated by AQP3 (Table 2), which is one member of the aquaporin (AQP) family and can promote the membrane exchange of water and regulate the osmotic balance [81]. The mRNA level of *GSK3B* is downregulated by many miRNAs through directly targeting 3’UTR of *GSK3B*, such as miR-19a/19b [82], miR-1246 [83], miR-1275 [84] and miR-582-3p [85] (Table 2). The protein levels of GSK3β is downregulated by lncRNA JPX [86] and PHLDA3 [87] and ARHGEF40 [88] (Table 2). JPX upregulated Twist1 by competitively sponging miR-33a-5p and subsequently induced EMT by activating Wnt/β-catenin signaling [86]. *PHLDA3* encodes a small 127 amino acid protein. *PHLDA3* is highly expressed in LUAD and is correlated with poor outcomes. PHLDA3 activates Wnt/β-catenin signaling through binding to GSK3β and promotes the oncogenic properties of NSCLC cells [87]. Ser 9 of GSK3β is the phosphorylation site for AKT, and the phosphorylation of this residue inactivates GSK3β. Recently, it was demonstrated that the scaffold protein AXIN allosterically protects GSK3β from phosphorylation at Ser9 by upstream kinases, which prevents accumulation of GSK3β phosphorylation (Ser9) in the Axin/GSK3β complex [89]. Thus, Ser9 phosphorylation of GSK3β does not affect Wnt/β-catenin signaling.

AXIN is another component of β-catenin destruction complex and contains two family members—AXIN1 and AXIN2. *AXIN1* expression was found to be downregulated in NSCLC by Zbed3 [90], GTPBP2 [91], YTHDF2 [23], APEX1 [92] and RIF1 [93] (Table 2). Zbed3 belongs to the family of BED‐zinc finger proteins and is overexpressed in NSCLC. Zbed3 enhances lung cancer development partially by inhibiting AXIN/GSK3β-mediated downregulation of β-catenin levels [90]. YTHDF2 is a reader of N6-methyladenosine (m6A) on RNA. *AXIN1* was a direct target of YTHDF2, which promoted *AXIN1* mRNA decay and subsequently activated the Wnt/β-catenin signaling [23]. APEX1 regulates aberrant alternative splicing of *AXIN1*. APEX1 expression was upregulated in NSCLC samples and reduced cell proliferation and induce apoptosis of NSCLC cells [92]. RIF1 promoted development and CSC-like properties of NSCLC through enhancing PP1-AXIN interaction and thereby activating Wnt/β-catenin signaling [93]. *AXIN2* expression was downregulated by a short peptide encoded by lncRNA DLX6-AS1, which is able to activate Wnt/β-catenin pathway in NSCLC cells [94]. MicroRNAs which downregulated APC expression include miR-4326 [95], miR-3607 [96] and miR-4739 [97] (Table 2). At the protein level, APC can be ubiquitinated by RNF115 and undergoes proteasomal degradation [98]. β-TrCP1 is an E3 ubiquitin ligase and one of the crucial components of β-catenin destruction complex. PKMYT1AR/miR-485-5p/PKMYT1 axis inhibited β-TrCP1 mediated ubiquitin degradation of β-catenin proteins, which in turn promote CSC maintenance and enhances tumorigenesis [99].

### β-Catenin

In NSCLC, β-catenin is found to be regulated at the transcriptional level, translational level, and through subcellular translocation. The mRNA expression level of *CTNNB1* can be upregulated by FLVCR1-AS1 [100], LINC01006 [101], lncRNA SNHG11 [102], TET [103], eIF3a [104], WSB2 [105], CIRP [106] and miR-214 [107] (Table 2). LINC01006 and lncRNA SNHG11 activate the Wnt/β-catenin pathway in LUAD cells by acting as sponges for miRNAs and elevating *CTNNB1* mRNA level [101, 102]. The TET family of DNA hydroxylases mediates the final DNA demethylation through sequential oxidation reactions, thus are key executors for maintaining a hypomethylated genome state [108]. Loss of TET reprograms Wnt/β-catenin signaling through impaired demethylation of Wnt antagonizing genes (e.g., *LRP4*, *CTNNBIP1*, *DACT1*, and *TMEM88*) to promote the development of NSCLC [103]. eIF3a and WSB2 regulate the transcription of *CTNNB1* [104, 105] (Table 2). CIRP (cold-inducible RNA binding protein) regulates *CTNNB1* mRNA expression level by binding its mRNA [106]. miR-214 directly targets 3′-UTR of *CTNNB1* to inhibit Wnt/β-catenin signaling in NSCLC cells [107].

The protein level of β-catenin in NSCLC cells is upregulated by LINC00514 [109], lncRNA ITGB1-DT [110], lncRNA UPLA1 [21], RNASEH1-AS1 [111], circEIF3I [112], circZSWIM4 [113], KDM2B [114], SETD1A [115], EHD1 [116], TRIM27 [117] and HORMAD1 [118] (Table 2). Of them, lncRNA ITGB1-DT facilitates LUAD progression through forming a positive feedback loop with ITGB1/Wnt/β-Catenin/MYC axis [110]. Desmoplakin has been found to inhibit Wnt/β-catenin signaling pathway in NSCLC [119]. LncRNA UPLA1 promoted Wnt/β-catenin signaling by binding to desmoplakin [21]. RNASEH1-AS1 exacerbated the progression of NSCLC by regulating the miR-516a-5p/FOXK1/β-catenin axis [111]. Circ-EIF3I could sponge miR-1253, which targets NOVA2 and promotes Wnt/β-catenin signaling [112]. CircZSWIM4 promotes the development of LUAD by targeting miR-370-3p and miR-873-5p to regulate FOXM1/β-catenin axis [113]. As the most well-characterized member of the mammalian C-terminal Eps15 homology (EH) domain-containing protein (EHD) family, EHD1 has been implicated in the resistance to EGFR-TKI in NSCLC through activation of PTEN/PI3K/AKT signaling [120]. Moreover, EHD1 activates a 14-3-3ζ/β-catenin/c-Myc regulatory circuit that synergistically promotes aerobic glycolysis in NSCLC [116].

The deubiquitination is closely related to Wnt/β-catenin pathway and that many regulators have been found to mediate the ubiquitination level of β-catenin, including lncRNA PKMYT1AR [99], FABP7 [121], and USP5 [122] (Table 2). Of them, the PKMYT1AR/miR-485-5p/PKMYT1 axis inhibits ubiquitin-mediated degradation of β-catenin, which in turn promotes CSC maintenance and enhances tumorigenesis in NSCLC [99]. FABP7 (fatty acid binding protein 7) is a cytoplasmic protein which is essential for lipid metabolism. FABP7 competitively inhibits the interaction between β-catenin and the components of its cytoplasmic destruction complex, thereby repressing the ubiquitination-mediated degradation of β-catenin [121]. USP5 encodes ubiquitin-specific peptidase 5, one of the deubiquitinating enzymes remove ubiquitin from target proteins. USP5 directly interacts with β-catenin, leading to deubiquitination, stabilization of β-catenin in NSCLC cells [122].

β-Catenin protein expression is also upregulated by FOXH1 [123], LRP8 [124], CBX4 [125], SMEK1 [126], JAML [127], ERCC6L [128], NOVA1 [129], SETDB1 [130], HMGB1 [131] and DEPDC1B [132] (Table 2), though the underlying molecular mechanism remains elusive. Additional regulators specifically mediate the level of active (i.e., unphosphorylated at Ser33/Ser37/Thr41) β‐catenin. Serine phosphorylation is necessary for recognition by the E3 ubiquitin ligase β-Trcp and subsequent proteasomal degradation. The active form of β-catenin is upregulated by MORC2 [133], PLAC8 [134], CCDC85B [135], KIF26B [136], TMED3 [137], ARHGEF40 [88] and tumor-intrinsic PD-L1 [138], with most of them modulated by AKT. Phosphorylation at Ser552 is able to regulate β-catenin activity. S100A4 is found to promote NSCLC tumor development through Wnt/β-catenin pathway-mediated autophagy inhibition. In this situation, S100A4 activates the Wnt/β-catenin pathway by the upregulation of the phosphorylation at Ser552 [139].

The nuclear accumulation of β-catenin is upregulated by lncRNA CBR3-AS1 [140], LINC00669 [141], MEF2D [142], ASNS [143], SRPK1 [144], Pygo2 [145], WDR74 [146], DSTN [147] and SOX9 [148] (Table 2). Of them, lncRNA CBR3-AS1 could physically interact with β-catenin and facilitate the activation of Wnt/β-catenin signaling thought promoting nuclear accumulation of β-catenin [140].

The complex of nuclear β-catenin and TCF4 transcription factor was upregulated by nuclear E-cadherin [149], Pygo1 [150], FOXP3 [151] and TRIB3 [152] (Table 2). β-catenin/TCF4 interaction was abolished by E-cadherin and was correlated with its nuclear localization, and consequently decreased β-catenin/TCF4 transcriptional activity. Subsequently, nuclear E-cadherin was a negative regulator of Wnt/β-catenin-elicited promotion of lung CSC phenotype [149]. FOXP3 can physically interact with TCF4 and β-catenin in the nucleus. High level of FOXP3 had a significant decrease in overall survival and recurrence free survival NSCLC patients [151].

## The negative regulators of Wnt/β-catenin signaling

Many negative regulators have been identified which act on Wnt ligands, receptors, components of β-catenin destruction complex, and β-catenin. The expression of these regulators might be achieved by aberrant expression, mutation, methylation, and histone modifications in NSCLC cells.

### Wnt ligands

Numerous negative regulators increase the transcriptional or protein level of multiple Wnt ligands, including WNT1 [153–155], WNT2B [156], WNT3A [157–159], WNT5A [160, 161], WNT5B [162] and WNT8B [160] (Table 3). *WNT1* expression was increased by miR-383 [153], miR-924 [155] and TMEM100 [154] (Table 3), whose expression was significantly decreased in NSCLC tissues and cells. MiR-383 regulates NSCLC cell proliferation by directly targeting *WNT1* [153]. MiR-924 blocked the progression of NSCLC by inhibiting RHBDD1/WNT1/β-catenin axis [155]. *WNT2B* was targeted by miR-577, which inactivated the Wnt/β-catenin pathway in NSCLC cells [156]. *WNT3A* expression was negatively regulated by circCCT3 [157], GPC5 [158] and GRIK3 [159] (Table 3). The expression of *WNT5A* and *WNT8B* was increased by miR-4757-3p in NSCLC cell lines [160]. The long isoform of *WNT5A* was targeted by miR-1253, which inhibited the proliferation and metastasis of NSCLC cells [161]. Likewise, WNT5B is negatively regulated by miR-5587-3p through binding to its 3′-UTR [162]. Taken together, these studies point to several miRNAs that function as tumor suppressors through inhibition of Wnt signaling.

**Table 3:** Negative Wnt regulators recently reported to involve in NSCLC

Name	Alterations	Target	Specimen	Clinical relevance	References
miR-383	Underexpression	*WNT1*	NSCLC tissues and cell lines	Unknown	[153]
miR-924	Underexpression	RHBDD1/Wnt1/β-catenin	NSCLC tissues and cell lines	TNM stage and lymph node metastasis	[155]
TMEM100	Histone deacetylase 6-mediated downregulation	WNT1/β-catenin	LUAD and LUSC from TCGA	Clinicopathological characteristics and poor OS	[154]
miR-577	Underexpression	3'-UTR of *WNT2B*	NSCLC tissues and cell lines	Tumor size and lymph node metastasis	[156]
circCCT3	N.D	3'-UTR of *WNT3A*	A549 cells	Unknown	[157]
GPC5	Promoter CpG methylation	Binding to WNT3A	LUAD tissues and cell lines	Poor prognosis	[158]
GRIK3	Underexpression	UBE2C and CDK1/WNT3A/β-catenin	NSCLC tissues	Poor prognosis	[159]
miR-4757-3p	Underexpression	3'-UTR of *WNT5A* and *WNT8B*	A549 cells	Unknown	[160]
miR-1253	Underexpression	3'-UTR of *WNT5A* (long isoform)	NSCLC tissues	TNM stages, Lymph node metastasis and poor OS	[161]
miR-5587-3p	N.D	WNT5B	LUAD tissues	Unknown	[162]
miR-3127-5p	Underexpression	FZD4	NSCLC metastatic tissues	Unknown	[163]
MITF	Overexpression	*FZD7* promoter	NSCLC tissues	Better OS and disease-free survival	[164]
lncRNA AK126698	Underexpression	*FZD8*	NSCLC tissues and cell lines	Tumor size and TNM stage	[165]
RASSF10	Underexpression	LRP6	Lung cancer specimens	Clinicopathologic characteristics and poor prognosis	[166]
LINC01089	Underexpression	miR-27a/SFRP1/β-catenin Axis	NSCLC samples	Clinicopathologic characteristics	[167]
miR-26a-5p	Underexpression	DNMT3A-Mediated SFRP1 Methylation	NSCLC tumor tissues	No significance in survival	[168]
Rab37	Underexpression	SFRP1-Wnt axis	NSCLC tissues	Clinicopathological characteristics and poor prognosis	[70]
hsa_circ_0006427	Underexpression	miR-6783-3p/DKK1 axis	LUAD tissues and cell lines	Clinicopathological characteristics and poor prognosis	[169]
hsa_circ_0018414	Underexpression	miR-6807-3p/DKK1 axis	LUAD tissues and cell lines	Poor prognosis	[22]
PCBP1	Underexpression	Stabilizing *DKK1* mRNA	LUAD tissues	Poor prognosis	[170]
CNN1	Underexpression	DKK1	LUSC tissues	Unknown	[171]
LINC00326	Underexpression	miR-657/DKK2 axis	NSCLC tissues and cell lines	Clinicopathological characteristics and poor prognosis	[172]
ARHGAP9	Underexpression	Transcription of *DKK2*	LUAD tissues	Poor prognosis	[173]
WWC3	Underexpression	DVL	NSCLC tissues and cell lines	Clinicopathologic characteristics and poor prognosis	[174]
circ-GSK3B (hsa_circ_0066903)	Underexpression	*GSK3B*	LUAD tissues	Unknown	[175]
HOXA4	Underexpression	Transcription of *GSK3B*	lung cancer tissues	Clinicopathologic characteristics and poor prognosis	[176]
DBH-AS1	Underexpression	miR-155/*AXIN1* axis	NSCLC tissues and cell lines	Unknown	[178]
RBM47	Underexpression	*AXIN1* mRNA stability via 3’-UTR binding	NSCLC tissues	Clinicopathologic characteristics and poor prognosis	[179]
LKB1	Mutation	Interacting with APC	N.D	N.D	[180]
FOXS1	Underexpression	APC	LUSC tissues	Unknown	[181]
miR-4429	Underexpression	*CTNNB1*	LUAD cells	Unknown	[182]
TMEM196	Underexpression	*CTNNB1* promoter	NSCLC tissues	Poor prognosis	[183]
SOX30	Underexpression	Transcription of *CTNNB1*	LUAD and LUSC tissues	Metastasis and poor prognosis	[184]
ARHGAP25	Underexpression	Transcription of *CTNNB1*	LUAD from TCGA	Tumor size and lymph node metastasis	[188]
EHMT2	Underexpression	Transcriptional activity of chromatin-bound* CTNNB1*	Mouse lung	Improved prognosis	[185]
LHX6	Underexpression	Transcriptionally silencing of *CTNNB1*	LUAD tissues	Clinicopathologic characteristics and poor OS	[186]
C/EBPα	Underexpression	Transcription of *CTNNB1*	LUAD tissues	Unknown	[187]
miR-214-3p	Underexpression	3’ UTR of FGFR1/β-catenin	FGFR1-amplified NSCLC from TCGA	Poor OS	[189]
miR-708-5p	Overexpression	DNMT3A/CDH1/β-catenin	NSCLC tissues	Better OS and lower recurrence	[260]
miR-520a	Underexpression	RRM2/β-catenin	NSCLC tissues	Poor prognosis	[190]
miR-34c-5p	Underexpression	TBL1XR1/β-catenin			[191]
miR-100	Underexpression	HOXA1/β-catenin	NSCLC tissues	Clinicopathologic characteristics and poor prognosis	[261]
miR-590	Underexpression	β-catenin	NSCLC tissues	Clinicopathologic characteristics and poor OS	[192]
circ-ITCH	Underexpression	β-catenin	Lung cancer tissues	TNM stages	[193]
circ-ZNF124	Overexpression	miR-498/YES/β-catenin axis	NSCLC tissues and cell lines	Unknown	[194]
DSTYK	Underexpression	N-terminal domain of β-catenin	Lung cancer tissues	Poor OS	[195]
EPB41	Underexpression	ALDOC/ β-catenin	NSCLC tissues	Poor prognosis	[196]
PJA1	Underexpression	FOXR2/ β-catenin	LUAD from TCGA	Poor OS	[197]
ZNF671	Underexpression	β-catenin	NSCLC tissues and cell lines	Poor prognosis	[198]
Shisa3	Underexpression	β-catenin degradation	NSCLC tissues and cell lines	Poor OS and progression-free survival	[200]
miR-489-3p	Underexpression	USP48/Ubiquitination of β-catenin	LUSC	Poor prognosis	[199]
ING5	N.A	Phosphorylation of β-catenin (Ser33/37)	A549 cells	Unknown	[201]
miR-147b	Underexpression	RPS15A/active β-catenin axis	NSCLC tissues	Unknown	[202]
EXT1	DNA methylation	Active β-catenin	NSCLC from TCGA	Better prognosis	[203]
KCTD11	Underexpression	Accumulation of nuclear β-catenin	NSCLC tissues	Clinicopathologic characteristics and poor prognosis	[204]
Fibulin-3	Underexpression	Nuclear β-catenin	NSCLC tissues and cell lines	Unknown	[205]
MARVELD3	Underexpression	Nuclear β-catenin	NSCLC tissues	Metastasis	[206]
RBM10	Underexpression	Nuclear β-catenin	LUAD tissues	Poor prognosis	[207]
SOX30	Underexpression	Competing with TCF for binding to β-catenin	Metastatic NSCLC tumors	Favorable independent prognostic biomarker	[208]
MYPT1	Underexpression	β-catenin/TCF4 complex	NSCLC tissues	Clinicopathologic characteristics and poor prognosis	[209]
IRF8	Promoter CpG methylation	TCF/LEF promoter	NSCLC tissues	Poor OS	[211]

*SCID* CB.17 severe combined immunodeficient-beige mice, *OS* overall survival

### Wnt receptors

Many negative regulators have been found to increase the expression or phosphorylation of Wnt receptors, including FZD4 [163], FZD7 [164], FZD8 [165] and LRP6 [166] (Table 3). miR-3127-5p increases the expression of *FZD4*, which promotes EMT and Wnt/β-catenin signaling in NSCLC [163]. MITF targets *FZD7* promoter, and silencing MITF can promote tumor cell migration, invasion and colony formation in LUAD cells [164]. LncRNA AK126698 targets *FZD8*, and downregulation of lncRNA AK126698 promotes the proliferation and migration of NSCLC cells through Wnt/β-catenin pathway [165]. For LRP5/6, the phosphorylation of LRP6 was decreased by RASSF10, and downregulation of RASSF10 promotes lung cancer proliferation and invasion [166]. *SFRP1*, highlighted above as a negative regulator of FZD family members, is itself negatively regulated by LINC01089 [167] and miR-26a-5p [168], and downregulation of LINC01089 and miR-26a-5p was found in NSCLC. Dysregulated Rab37-SFRP1 pathway confers NSCLC stemness via the activation of Wnt/β-catenin signaling. Rab37 expression positively correlates with SFRP1 level in NSCLC patients and negatively correlated with tumor stage of NSCLC [70]. Expression of *DKK1*, previously discussed as a negative regulator of LRP5/6 coreceptors, was upregulated by* hsa_circ_0006427* [169], *hsa_circ_0018414* [22], PCBP1 [170] and CNN1 [171] (Table 3). *DKK2* expression was found to be increased by LINC00326 [172] and ARHGAP9 [173]. Signal transducer DVLs were negatively regulated by the scaffolding protein WWC3 [174]. WWC3 interacts with DVLs, prevents casein kinase 1ϵ from phosphorylating DVLs, and inhibits the nuclear translocation of β-catenin, and downregulation of WWC3 was found in NSCLC [174].

### β-Catenin destruction complex

Many negative regulators exert their inhibitory effects on components of the β-catenin destruction complex by regulating transcriptional activity, mRNA stability, and protein expression. GSK3β expression can be increased by *circ-GSK3B* [175] and HOXA4 [176] (Table 3). *circ-GSK3B* competitively sponges miR-3681-3p and miR-3909, leading to elevated *GSK3B* expression [175]. HOXA4 belongs to the Homeobox (HOX) gene family, which encode transcription factors that control cell differentiation and embryonic development [177]. HOXA4 significantly increased *GSK3B* expression by binding its promoter region and promoting its transcription [176]. *AXIN1* expression was regulated by lncDBH-AS1 [178] and RBM47 [179] (Table 3). Silence of lncDBH-AS1 enhances proliferation of NSCLC cells by activating Wnt signaling pathway via the miR-155/AXIN1 axis [178]. The RNA-binding protein RBM47 inhibits the metastasis of NSCLC through modulation of *AXIN1* mRNA stability [179]. APC was regulated by LKB1 [180] and FOXS1 [181] (Table 3). LKB1 binds to APC to suppress the Wnt/β-catenin signaling pathway [180]. FOXS1 inhibits Wnt/β-catenin signaling pathway by increasing APC expression in LUSC cells [181].

### β-Catenin

Multiple negative regulators act on β-catenin by repressing its transcriptional expression, protein level, and nuclear accumulation. The mRNA expression level of *CTNNB1* was found to be suppressed by miR-4429 [182], TMEM196 [183], SOX30 [184], EHMT2 [185], LHX6 [186], C/EBPα [187] and ARHGAP25 [188] (Table 3). The downregulation of these negative regulators was found in NSCLC tissues and cell lines (Table 2). LHX6 suppressed the Wnt/β-catenin pathway through silencing the transcriptional expression of *CTNNB1*. LHX6 expression was found to be a favorable independent prognostic factor for overall survival (OS) of LUAD patients and clinical characteristics [186].

The protein level of β-catenin was negatively regulated by miR-214-3p [189], miR-708-5p, miR-520a [190], miR-34c-5p [191], miR-100, miR-590 [192], cir-ITCH [193], circ-ZNF124 [194], DSTYK [195], EPB41 [196], PJA1 [197] and ZNF671 [198] (Table 3), and downregulation of these regulators was found in NSCLC. MiR-590 was down-regulated in NSCLC tissues and cell lines, and inhibited the Wnt/β-catenin pathway in NSCLC cells [192]. Interestingly, it was also found that miR-590 was negatively correlated with YAP1 expression NSCLC tumor tissues, and miR‑590 suppressed YAP1 expression by targeting its 3’ UTR in NSCLC cells [192]. circ-ZNF124 regulated *YES1* expression by acting as a sponge of miR-498, thus restraining NSCLC development by suppressing Wnt/β-catenin signaling pathway [194]. DSTYK encodes dual serine/threonine and tyrosine protein kinase which phosphorylated the N-terminal domain of β-catenin and inhibited Wnt/β-catenin signaling, leading to the inhibition of tumorigenesis in a LUAD mouse model [195]. EPB41 forms a complex with ALDOC, leading to disassociation of the β-catenin destruction complex, reduced proteasomal degradation of β-catenin, elevated cytoplasmic accumulation, and nuclear translocation of β-catenin [196].

Multiple regulators inhibit Wnt/β-catenin signaling through regulating the ubiquitination-mediated degradation of β-catenin, including miR-489-3p [199], Shisa3 [200] and ING5 [201] (Table 3). miR-489-3p hampers the progression of NSCLC through targeting USP48 to increase the ubiquitination of β-catenin [199]. Shisa3 accelerates the degradation of β-catenin through decreasing the availability of FZDs [200]. ING5 overexpression promotes phosphorylation of β‐catenin at Ser33/37, leading to a decreased β‐catenin protein level [201]. The protein level of β‐catenin could also be manifested as active β‐catenin (unphosphorylated at Ser33/Ser37/Thr41), which is negatively regulated by miR-147b [202], and EXT1 [203].

The accumulation of nuclear β-catenin is negatively regulated by KCTD11 [204], Fibulin-3 [205], MARVELD3 [206] and RBM10 [207] (Table 3), and underexpression of these regulators was found in NSCLC. KCTD11 inhibits progression of lung cancer by binding to β-catenin [204]. MARVELD3 (MAL and relevant proteins for vesicle trafficking and membrane link domain 3) is a tight junction protein which influences EMT. Lower protein levels of MARVELD3 were observed in NSCLC samples, and associated with tumor metastasis. Mechanistically, MARVELD3 inhibits TGF-β1 induced EMT by suppressing Wnt/β-catenin signaling in NSCLC cells [206].

The interaction between nuclear β-catenin and TCF4 transcription factor was suppressed by SOX30 [208] and MYPT1 [209] (Table 3). SOX30 attenuates Wnt/β-catenin signaling via directly repressing the transcription of β-catenin or competitively binding to β-catenin [208]. SOX30 also suppresses Wnt/β-catenin signaling pathway through upregulation of desmosomal genes including DSP and JUP [210]. The TCF/LEF transcription factor was regulated by IRF8, which repressed β-catenin nuclear translocation and its activation [211].

## Wnt/β-catenin signaling impacts therapeutic sensitivity and resistance of NSCLC

The abnormal activation of Wnt components and regulators influences response to several therapies for NSCLC, including targeted therapy, radiotherapy and chemotherapy.

### Targeted therapy

In EGFR-mutant NSCLC, *FOXM1* rs3742076_G (rs3742076) was found to confer gefitinib resistance by increasing FOXM1 protein stability through activating Wnt/β-catenin signaling pathway [20] (Table 4). Wnt inhibitory factor-1 (WIF1) is a secreted antagonist of Wnt/β-catenin signaling and binds to Wnt ligands extracellularly [68]. The status of *WIF1* methylation is associated with progression free survival [212] and gefitinib response [213], possibly through regulation of this Wnt-FOXM1 axis. FLNA and ANXA2 cooperatively promotes the activation of the Wnt/β-catenin pathway, which contributes to gefitinib resistance [214]. DCLK1 expression confers EGFR-TKI resistance to LUAD through regulating Wnt/β-catenin activity [215]. Lower *LHX6* expression was detected in HCC827/ER cells and re-expression of *LHX6* increased erlotinib sensitivity through activating Wnt/β-catenin signaling [216]. The neurotransmitter acetylcholine (ACh) was specifically accumulated in drug-tolerant persister (DTP) cells. The upregulated ACh metabolism mediated EGFR-TKI sensitivity partially through activating Wnt/β-catenin signaling [217]. Multiple negative regulators were found to promote the activation of Wnt/β-catenin signaling in NSCLC. The expression of circFBXW7 was found to significantly downregulated in osimertinib-resistant cell lines (Table 4). circFBXW7 resensitizes resistant LUAD cells to osimertinib. Mechanistically, circFBXW7 encodes a short polypeptide, which directly interacts with β-catenin. This interaction leads to reduced stability of β-catenin by inducing ubiquitination, thereby attenuates Wnt/β-catenin signaling [218]. Other targeted therapies may also be susceptible to Wnt signaling. For example, case-level data exists showing a secondary *CTNNB1* mutation correlating with failure of ALK TKIs [219].

**Table 4:** Wnt regulators recently reported to associate with the therapeutic sensitivity and resistance of NSCLC

Name	Alterations	Target	Treatment	Specimen	Clinical effect	References
SRPK1	Overexpression	β-catenin/EGFR	Gefitinib	Advanced NSCLC	Poor PFS	[262]
FOXM1	rs3742076_G	Nuclear β-catenin	Gefitinib	NSCLC patients	Poor prognosis	[20]
FLNA and ANXA2	Overexpression	β-catenin	Gefitinib	PC-9, HCC827 and H3255	Resistance	[214]
DCLK1	Overexpression	Cytoplasmic β-catenin	Gefitinib, osimertinib	LUAD tissues	Poor prognosis	[215]
LHX6	Underexpression	Nuclear β-catenin	Erlotinib	HCC827 and HCC827/ER cells	Resistance	[216]
ACh	Overexpression	Wnt ligands	Osimetinib	NSCLC patients	Worse drug response	[217]
circFBXW7	Underexpression	β-catenin	Osimertinib	HCC827 and H1975 cells	Resistance	[218]
Exosomal TPX2	Overexpression	β-catenin	Docetaxel	NSCLC patients	Poor prognosis	[225]
RRM2	Overexpression	β-catenin	Cisplatin	Cisplatin-resistant A549/DDP cell line	Clinicopathologic characteristics and poor prognosis	[223]
miR-32 and miR-548a	Underexpression	ROBO1/nuclear β-catenin	Cisplatin	DDP-resistant NSCLC tissues	Poor prognosis	[226]
miR-181c	Overexpression	WIF1	Cisplatin	NSCLC tissues	DDP sensitivity	[227]
CAFs	NA	HK2/β-catenin	Radiation	NSCLC patients	Radioresistance	[230]
UBE2T	Overexpression	FOXO1/GSK3β/β-catenin	Radiation	NSCLC tissues	Radiation resistance	[232]

*CAFs* cancer-associated fibroblasts, *PFS* progression-free survival

### Chemotherapy

Cisplatin (DDP) is the most widely used chemotherapeutic agent for NSCLC [220, 221]. *DVL2* overexpression was found in DDP-resistant NSCLC A549 (A549/DDP) cells compared to the parental A549 cells (Table 4). Inhibition of DVL2 resensitizes DDP-resistant NSCLC cells through downregulating Wnt/β-catenin signaling [222]. RRM2 is a component of ribonucleotide reductase. Higher levels of *RRM2* expression was found in A549/DDP cells. Knockdown OF *RRM2* promoted the sensitivity of A549/DDP cells to cisplatin through Wnt/β-catenin signaling pathway [223]. TPX2 is a microtubule-related protein in mobile mitosis and spindle assembly [224]. Transmission of exosomal TPX2 promotes the resistance of NSCLC cells to docetaxel through increasing the protein level of β-catenin [225]. Many miRNAs have been found to involve in chemotherapy of NSCLC. miR-32 and miR-548a were poorly expressed in DDP-resistant NSCLC, re-expression of miR-32 and miRNA-548a promotes the sensitivity of NSCLC cells to cisplatin by targeting ROBO1/β-catenin axis [226]. miR-181c expression was upregulated in DDP-resistant NSCLC cells, and miR-181c negatively regulated *WIF1* expression through directly binding to WIF1 (Table 4) [227].

### Radiotherapy

Wnt/β‑catenin signaling has been found to associate with radiotherapeutic sensitivity and resistance of NSCLC. WNT5A expression is often upregulated in radiation-resistant NSCLC cells (Table 4). Mechanistic investigation indicated that altered WNT5A expression affects radiosensitivity of NSCLC via Wnt/β-catenin pathway [228]. Disabled-2 (Dab2) is known as a tumor suppressor and Wnt pathway inhibitor. It has been found that promoter de-methylation of Dab2 gene enhances X-Ray irradiation sensitivity of NSCLC cells [229]. Cancer-associated fibroblasts (CAFs), one main component of the tumor microenvironment, regulated DNA damage response of NSCLC cells following irradiation. Mechanistically, CAFs up-regulate and stabilize c-Myc, leading to the transcription activation of HK2 kinase, a key rate-limiting enzyme in glycolysis by activating Wnt/β-catenin pathway [230]. Therefore, CAFs contribute to the radioresistance of NSCLC cells by promoting the glycolysis in a Wnt/β-catenin signaling-dependent manner. UBE2T has been found to promote NSCLC progression [231]. Recently, it was found that UBE2T promotes radioresistance in NSCLC (Table 4). Mechanistically, UBE2T promotes EMT partially through Wnt/β-catenin signaling activation [232]. Therefore, Wnt/β-catenin signaling might be a potential target for enhancing radiotherapy sensitivity.

### Immunotherapy

Aberrant activation of Wnt/β-catenin signaling promotes the escape of cancer cells from immune surveillance, inhibits T-cell infiltration, and mediates the response to immunotherapy [233, 234]. It has been shown that WNT1 silences chemokine genes in dendritic cells and induces adaptive immune resistance in LUAD [235]. Tumor β-catenin expression is associated with immune evasion in NSCLC with high tumor mutation burden [236]. By bioinformatic analysis, *DKK1* was identified as a candidate gene related to composition of tumor immune microenvironment and response to immunotherapy in LUAD patients [237]. Therefore, Wnt/β-catenin pathway might be a potential mechanism involved in the regulation of response to immunotherapy.

## Potential NSCLC treatments through suppression of Wnt/β‑catenin signaling

Multiple small molecules exist which inhibit positive Wnt regulators, providing an avenue to suppress Wnt/β‑catenin signaling in NSCLC. Porcupine protein, a membrane bound O-acetyltransferase, regulates the biogenesis of Wnt ligands. The Porcupine inhibitor LGK‑974 functions by binding to Porcupine and competing with acyl-CoA, thus blocking Wnt acetylation by Porcupine and inhibiting Wnt/β‑catenin signaling [238] (Table 5). LGK-974 modifies tumor‑associated macrophages resulting in inhibition of NSCLC cells [239], with one Phase 1 study still active (NCT01351103).

**Table 5:** Agents recently reported to inhibit the development and therapeutic response of NSCLC through regulating Wnt/β-catenin signaling

Name	Target	Phenotype	Models	References
LGK‑974	Porcupine	Tumor‑associated macrophages	A549 and H1299 cells	[239]
NCT-80	Akt/ERK/STAT3/Wnt ligands	Viability and migration	LLC-Luc allograft model	[240]
WP1130	USP5/β-catenin	Sphere formation, migration, and invasion	CL1-5 cells and LIJ cells	[122]
Triptolide	Wnt inhibitory factors (WIF1, FRZB, SFRP1, ENY2, and DKK1)	Tumor growth and metastasis	A549, H460 cell lines and CDX mouse model	[263]
Triptolide	p70S6k/GSK3β/β-catenin	EMT	Taxol-resistant A549 cells and CDX mouse model	[242]
Triptolide derivative MRx102	WIF1	Tumor formation and metastasis	PDX mouse model	[244]
Ethacrynic acid	p-LRP6/nuclear β-catenin	Antitumor effects of afatinib	EGFR L858R/T790M-mutated NSCLC cells	[245]
IMU1003	β-catenin	GR cell viability	Gefitinib-resistant PC-9 cells (GR cells)	[246]
SHH002-hu1	Fzd7	Migration and invasion	Fzd7 + NSCLC tissues and cell lines (A549 and H1975)	[247]
Berberine nanostructures	β-catenin	Unknown	A549 cells	[254]

Similarly, NCT-80 is an Hsp90 inhibitor which upregulates the transcription of Wnt ligands through Akt- and ERK-mediated activation of STAT3 (Table 5). NCT-80 effectively overcomes acquired resistance to chemotherapy and EGFR targeting anticancer therapy by inducing apoptosis and inhibiting EMT [240]. USP5 has also been found to be a positive regulator of Wnt/β‑catenin signaling in NSCLC. Targeting USP5 with the small molecule WP1130 induced the degradation of β-catenin, and showed markedly inhibitory effects on tumor growth and metastasis [122].

Many natural compounds inhibit the development of NSCLC through targeting Wnt/β-catenin signaling. Triptolide is a natural component extracted from *Tripterygium wilfordii*, a Chinese plant (Table 5). Triptolide inhibits EMT phenotype in both gefitinib-resistant [241] and taxol-resistant LUAD [242], possibly through the p70S6k/GSK3β/β-catenin signaling pathway [242], though its clinical applications are limited by severe hepatotoxicity [243]. Similarly, the triptolide derivative MRx102 significantly inhibited NSCLC proliferation through upregulating WIF1, a well-recognized negative regulator targeting Wnt ligands (Table 5) [244].

Many chemicals can inhibit the development and therapeutic resistance of NSCLC by targeting Wnt/β-catenin signaling. Ethacrynic acid, a loop diuretic, suppresses EMT of A549 cells via blocking of NDP-induced Wnt signaling [245] (Table 5). IMU1003 is an atrarate derivative which dramatically decreased the emergence of osimertinib-resistant colonies through inhibiting the nuclear localization of β-catenin [246] (Table 5).

FZD receptors are perhaps the best-validated Wnt regulators as therapeutic targets. Preclinically, SHH002-hu1 is an FZD7-targeting antibody which specifically binds *FZD7*-expressing NSCLC tissues and cells (Table 5). SHH002-hu1 effectively inhibits the migration and invasion of NSCLC cells by suppressing the activation of Wnt/β-catenin signaling [247, 248]. Three anti-FZD agents—OTSA101, vantictumab (OMP-185R), and ipafricept (OMB-54F28)—have entered clinical study. OTSA101 is an anti-FZD10 monoclonal antibody, and is radiolabeled to achieve an antiproliferative effect. OTSA101 has been studied clinically with indium 111 and yttrium 90 (NCT04176016 and NCT01469975) [249], and with actinium 225 preclinically [250]. Vantictumab is likewise a monoclonal antibody, binding to FZD1, 2, 5, 7, and 8, and has been studied clinically for a variety of cancers, including lung cancer (NCT01973309, NCT01957007, and NCT02005315) [251]. Lastly, ipafricept is a FZD8 “decoy” receptor, a truncated FZD8 protein fused to the Fc region of human IgG1 [252]. This decoy presumably functions by sequestering Wnt ligand, thus dampening canonical Wnt signaling. A total of four clinical studies of ipafricept have been completed with no results posted yet (NCT02069145, NCT02092363, NCT02050178, and NCT01608867).

Antisense oligonucleotide (ASO) drugs have been reported to be effective at inhibiting tumor growth both in vitro and in vivo (Table 5) [253]. LncRNA PKMYT1AR promotes CSC maintenance in NSCLC via activating Wnt signaling pathway. PKMYT1AR targeting ASO was found to dramatically inhibit tumor growth in vivo [99].

Nanoparticle formulations can improve the efficacy of existing drugs. Berberine, an isoquinoline alkaloid known for its anti-cancer and anti-inflammatory properties, shows low solubility and bioavailability (Table 5). The physiochemical functions of berberine can be largely improved by being encapsulated into liquid crystalline nanoparticles. Berberine liquid crystalline nanoparticles significantly suppresses the expression of β-catenin at both transcription and translation level [254].

## Conclusions

Recent identification of multiple Wnt regulators, and their dysregulation in NSCLC, emphasize the importance of Wnt/β-catenin signaling in NSCLC development and therapeutic response. These regulators act on Wnt ligands, receptors, signal transducers, and transcriptional effectors, as well as those well-known regulators. Dysregulation of these Wnt regulators can be either genetic or epigenetic, resulting in overexpression, underexpression, or gain of function and loss of function. Multiple circRNAs and micropeptides have been found to regulate Wnt/β-catenin signaling in NSCLC. Continued study of these regulators improves our understanding of NSCLC biology and may open avenues to novel therapies through the direct targeting of Wnt/β-catenin signaling.

### Supplementary Information

Supplementary Material 1

## Data Availability

The authors declare that all data supporting the findings of this study are provided in the Supplementary Data file. The GENIE Cohort v14-public dataset is publicly available through Sage Bionetworks (https://www.aacr.org/professionals/research/aacr-project-genie/aacr-project-genie-data/).
